# Parametric Optimization of Torsional Parameters of Ferrocement “U” Wrapped Beams Using Recent Meta-Heuristic Optimization Algorithms

**DOI:** 10.3390/ma16206727

**Published:** 2023-10-17

**Authors:** Gopal Charan Behera, Dilip Kumar Bagal, Praddyut Kumar Muduli, Louai A. Maghrabi, Harish Chandra Mohanta

**Affiliations:** 1 Department of Civil Engineering, Government College of Engineering, Kalahandi 766003, Odisha, India; pradyut.muduli@gmail.com; 2 Department of Mechanical Engineering, Government College of Engineering, Kalahandi 766003, Odisha, India; dilipbagal90@gmail.com; 3 Department of Software Engineering, College of Engineering, University of Business and Technology, Jeddah, Saudi Arabia; l.maghrabi@ubt.edu.sa; 4 Department of Electronics and Communication Engineering, Centurion University of Technology and Management, Jatni 752050, Odisha, India; harishmohanta@cutm.ac.in

**Keywords:** ferrocement “U” wrap, WASPAS, MARS, regression analysis, torque, twist

## Abstract

Structural elements are subjected to different types of loads, one of which is a torsional load. Due to the complexity of the analysis, torsion was not given much importance in earlier days. With stringent updates in codal provisions and due to architectural modifications, torsion is now considered one of the major parameters for structural design. The main aim of this paper is to analyze distressed elements due to torsion. It highlights different approaches, such as destructive and non-destructive processes, to be adopted to estimate the torsional parameters of a ferrocement “U” wrapped beam. The destructive method is the experimental determination of parameters, which is absolutely necessary. The non-destructive method includes an analytical method based on a softened truss model as well as a soft computing method. The soft computing method is based on the regression coefficient analysis method along with two recent optimization algorithms, i.e., (1) ARO (artificial rabbits optimization) and (2) DAOA (dynamic arithmetic optimization algorithm). The predicted results are found to be in agreement with the experimental values (destructive method). Lastly, the obtained results from both proposed methods are analyzed, and it is found that both algorithms can be utilized in any engineering problem to determine the global optimum value with corresponding input optimal settings. As the experimental method is time-consuming and expensive, analytical, and soft computing methods can be preferred over the experimental method.

## 1. Introduction

In each part of the globe, concrete is found to be the most extensively used construction material. Concrete structures are subjected to different loads such as shear, bending, axial tension, compression, or a combination of all these loads. A beam that has been subjected to torsional loading must determine torsional characteristics like torque and twist. There are two categories for figuring out a structural element’s torsional strength. Using torsional loading to test an element and determine its maximum strength is one method. The alternative is to use non-destructive technologies to forecast the ultimate torsional strength of structural components.

The absence of theories to quantify concrete torsional strength and the assumption that sections designed for shear and flexure would take care of torsional strength kept torsion in the back seat of design until the nineteenth century. Calamities of nature and artificial havoc caused by human society are the main threats to structures. Instant changes in stress levels in structures caused by natural disasters and havoc cause damage that necessitates upgrading rather than demolition.

Ferrocement-wrapped “U” beams are structural members composed of a steel frame embedded in mortar and reinforced with wire mesh. They are used in many different types of construction projects, including bridges, buildings, and walls. The unique shape of the “U”-wrapped beams allows them to provide additional strength and stiffness to the structure while reducing its overall weight. Fortifying distraught structures is necessary to bear the loads and meet recent codal provisions. FRP wrapping is expensive and requires skilled workmanship, making it popular among high-income groups or areas [1]. The strength of concrete structures may be increased through FRP wrapping [2,3]. Instead of FRP, a ferrocement wrap is a superior option for underdeveloped nations. Different wrapping techniques are needed for various repair materials. The wrapping technique must be decided upon based on the wrapping material. According to [4], the wrapping technique is determined by the kind of material, the cost of wrapping, and the accessibility of repair resources and people. Wrapping a weak component with FRP might increase its strength. When FRP is wrapped, strength increases significantly, and the mechanism of failure of sections changes as well [5]. A portion becomes stronger if it is completely wrapped. The ability to wrap a whole area is not always practical [6,7]. The usage of “U” wraps is often thought to be better. Studies have been carried out to measure the torque and twist of FRP wraps on all four sides [8]. Chalioris (2008) [9] concentrated on strengthening “U” wraps. According to [10], ferrocement wrapping has the capacity to increase strength. By considering cost and strength, ferrocement may be used in lieu of FRP as a wrapping material. Iron gives attributes including waterproofing and strength in tension, compression, and bending that are superior to those of concrete [11]. A high volume of small diameter and closely spaced wires in ferrocement along longitudinal and transverse directions improves many engineering properties of mortar, including tensile, compressive, toughness, and flexural strength [12]. The combined action of mortar and steel results in cracking. Beyond cracking, steel becomes more active than mortar. Ferrocement can be treated as a composite, where the mortar serves as the matrix phase and wire meshes as the reinforcement and can be a substitute for FRP in developing countries. A mortar matrix is used to join smaller diameter wires on both sides of a mesh in two perpendicular directions to generate ferrocement. The added reinforcement, which is distributed evenly throughout the whole surface, increases strength. Cement wrap has been shown to be waterproof and resistant to corrosion and sulfate attack. Structures made with ferrocement may stop fractures before they start [13]. Stirrups with a “U” form can increase the strength and stiffness of beams, as shown by [14]. There is sufficient evidence in this body of research to support the improvement of strength and stiffness of “U” wraps. Peripheral concrete cover is a highly stressed region under torsional loads. Hence, any substitution in this region with a high-strength material enhances the torsional strength noticeably. The above-mentioned research works for wrapping materials, either full wrap or “U” wrap, have shown improvements in strength and ductility for distressed beams subjected to torsion. On the torsional behavior of reinforced concrete beams with Ferrocement “U” wraps for many potential scenarios of reinforcement, no soft computing study has been documented. The investigation’s broad aims are as follows:

- To measure the torsional characteristics of “U” wrapped beams at the cracking and final phases, develop a soft computing model.
- Compare the torsional capabilities of reinforced concrete beams with “U” wraps to soft computing values obtained by experimentation and analysis.

## 2. Different Approaches to Predict Torsional Parameters

All of these methods have their own merits and demerits. Analytical methods are often fast and inexpensive but may not be very accurate for complex structures. Numerical methods can be more accurate but can be time-consuming and computationally expensive. Experimental methods provide direct measurement of torsional properties but are limited by the range of conditions that can be tested in a laboratory setting and can be expensive and time-consuming to conduct. The choice of method often depends on the specific requirements of the design problem and the available resources.

### 2.1. Experimental Approach

**Figure 1:**
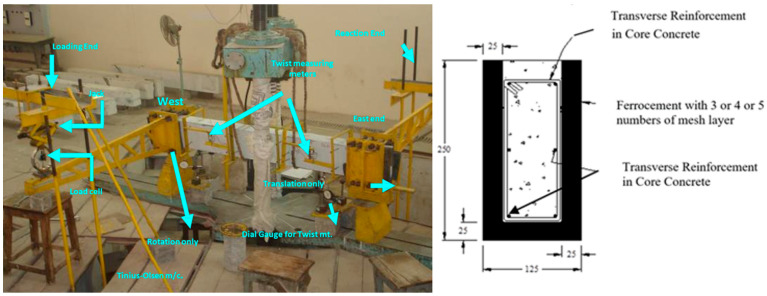
Experimental set-up for torsion testing and beam cross-section.

The best method for determining the torsional strength of ferrocement “U” wrapped beams is to cast prototype beams and put them through torsional loading tests. All of the 2000 mm long, 125 mm wide, and 250 mm deep beams were cast in one piece. The primary changes were the quantity of reinforcement in the core concrete, the grade of the core concrete, and the number of mesh layers in the outside perimeter. To push the failure towards the core area of 1500 mm, which can contain two spirals of fractures, end sections of 250 mm were strengthened further. The beam is either singularly reinforced, under-reinforced, over-reinforced in the longitudinal or transverse direction, or completely over-reinforced when there is reinforcement in the core. The degree of twisting is determined by the quantity of reinforcement applied either longitudinally or transversely [15]. The M35 grade concrete and M40 grade mortar were used to cast all of the normal-strength concrete beams, while M60 grade concrete and M55 grade mortar were used to produce the high-strength concrete beams. An amount of 0.72 mm mesh with a yield strength of 250 MPa was employed in the cement zone. Abbreviations for longitudinally under-reinforced, transversely over-reinforced, and totally over-reinforced are U, L, T, and C, respectively. A plain beam designated as PN4 has a core of M35 grade concrete, mortar with a compressive strength of 40 MPa, and four layers of mesh in the ferro-concrete zone without any reinforcing. A beam made of M35 grade concrete, M40 grade mortar, and three different mesh layers is referred to as CoN4 in the ferrocement zone. LoH4 is a beam that has been longitudinally reinforced with M60 grade core concrete, M55 grade mortar, and four mesh layer numbers in the periphery [15]. Figure 1 shows the type of testing that is performed to determine the strength and structural integrity of beams under various loads and forces. The experimental set-up typically involves a beam sample that is fixed at both ends and loaded with a weight or torque to simulate real-world conditions. The beam is then observed for any signs of deformation, cracking, or failure. By measuring the beam’s response to the applied load, engineers can determine the beam’s load-carrying capacity and assess its suitability for a particular application. This information is then used to improve the design of the beam and ensure its structural stability in real-world conditions.

### 2.2. Analytical Model

Determination of torsional parameters of ferrocement “U” wrapped beams by an experimental method involves the destruction of prototype structures and provides parameters related to that particular beam. According to the experimental method, the strength of a beam cannot be predicted if beam dimensions, or material properties change. So, the analytical method is employed to predict the same. The analytical method is based on Hsu’s softened truss model with some modifications to the material properties. A wrapped beam is characterized by three stages: the elastic stage, the cracking stage, and the post-cracking stage. Before the initiation of a micro-crack, the torque-twist diagram is linear. The linearity ends once the micro-crack is initiated, and the reinforcement is activated. It is in between micro-cracking and macro-cracking. When the shear stress in the wrapping equals the cement’s cracking strength (considering the mortar’s tensile strength as well as that of the mesh reinforcement), the micro-cracking stage is completed. The post-cracking stage begins after the micro-cracking stage. Different equations need to be developed for each of these three steps. The elastic stage continues until the shear stress introduced on either the longer or shorter side matches, respectively, the shear stress of the concrete matrix or mortar matrix. When the induced shear stress equals the shear stress of mortar or concrete, taking reinforcement contribution into account, the micro-cracking stage is complete. The detailed procedure has been described by the author [15].

### 2.3. Soft Computing Method

Soft computing encompasses a range of computational techniques, including fuzzy logic, neural networks, evolutionary algorithms, and various machine learning methods. These approaches excel in tackling complex problems involving uncertain or “noisy” data. They find applications in robotics, image processing, natural language processing, and other AI-related domains. Through soft computing methods (SCMs), computers can learn patterns or rules from existing data, such as field instrumentation or case studies.

These SCMs encompass a diverse set of techniques, including multivariate adaptive regression splines (MARS), artificial neural networks (ANNs), support vector machines (SVMs), decision trees (DT), gradient boosting machines (GBM), logistic regression (LR), Gaussian processes (GP), and hybrid models like the adaptive neuro-fuzzy inference system (ANFIS), gene expression programming (GEP), and weighted aggregated sum product assessment (WASPAS). Additionally, methods like random forests and Gaussian processes fall under the umbrella of soft computing techniques [16].

#### 2.3.1. Regression Analysis Method

A statistical technique for analyzing data and determining the relationships between variables is regression analysis. Based on the values of one or more predictor variables (the independent variables), it is used to predict the value of a response variable (the dependent variable). Machine learning applications often utilize regression analysis to find patterns in data and forecast future values. Regression analysis is used to forecast the torque and twist of ferrocement “U” wrapped beams. Regression analysis is a statistical method for describing how two independent variables are related to one another and how much one variable changes when the other one does. The mathematical technique of least squares is used to estimate the parameter coefficient. Because of their ease of use and useful interpretation, regression methods are crucial tools.

##### Simple Linear Regression

A form of regression analysis called simple linear regression uses a linear equation to represent the relationship between two variables. It is used to forecast one variable’s value in light of another’s value. The linear equation may be used to forecast future values or to clarify how the two variables are related. To put it another way, if X and Y are two variables that are connected to one another, then linear regression methods may be used to estimate the value of Y for a given value of X. Calculate the value of X based on the value of Y.

A statistical technique known as single regression models the connection between one independent variable and one dependent variable. Based on the value of the independent variable, it is used to forecast the value of the dependent variable. For instance, depending on a person’s height, weight may be predicted using a single regression. The formula would be as follows:

Weight = a + b × (Height)(1)

where a and b are constants determined through regression analysis, weight is the dependent variable, and height is the independent variable.

Multiple regression, on the other hand, is a statistical method that models the relationship between a dependent variable and multiple independent variables. It is used to predict the value of the dependent variable based on multiple independent variables. For example, a multiple regression could be used to predict the salary of a person based on their education level, years of experience, and job title. The equation would look like the following:

Salary = a + b1 × (Education) + b2 × (Experience) + b3 × (Job Title)(2)

where a, b1, b2, and b3 are constants determined through regression analysis, salary is the dependent variable, and education, experience, and job title are the independent variables.

A single regression is used to model the relationship between one independent variable and one dependent variable, while multiple regression is used to model the relationship between multiple independent variables and one dependent variable [17].

## 3. Theory of Optimization Approaches

The theory of optimization is the mathematical study of decision-making processes that aim to maximize or minimize a given objective function. It is a branch of mathematics that deals with finding optimum solutions to problems that involve maximizing or minimizing certain objectives over a set of constraints. The theory of optimization is used in many disciplines, including economics, engineering, operations research, and computer science. Optimization is needed in civil engineering to improve the efficiency and effectiveness of the designs. Optimization techniques can be used to find the most cost-effective and structurally sound design for a given project. Optimization can also be used to identify the most efficient material usage, analyze the structural behavior of a system, and minimize the risk of failure. Here, two recent nature-based optimization techniques are utilized, such as ARO (artificial rabbits optimization) and DAOA (dynamic arithmetic optimization algorithm), respectively.

### 3.1. Artificial Rabbits Optimization

The artificial rabbits optimization (ARO) algorithm was first proposed by a group of researchers from the University of Tehran in 2019. The algorithm is inspired by the behavior of rabbits in nature and is designed to improve the efficiency of search algorithms such as particle swarm optimization. ARO (artificial rabbits optimization) leverages actual rabbits’ foraging and hiding techniques, as well as their energy shrink, to transition between the two. It is a contemporary bio-inspired meta-heuristic optimization approach for tackling engineering challenges. The artificial rabbits optimization (ARO) algorithm has been used to solve a variety of optimization problems, including scheduling and routing, image processing, and robotics [18]. It has also been used to optimize parameters in machine learning and artificial intelligence models. The ARO algorithm has been found to be effective at finding high-quality solutions quickly, making it an attractive option for many types of optimization problems.

Adult rabbit update equation:

X_a(t + 1) = X_a(t) + alpha × (X_best(t) − X_a(t)) + beta × (X_a(t) − X_worst(t))(3)

where

Xa(t) is the position of the adult rabbit at time t;
Xbest(t) is the position of the best rabbit in the population at time t;
Xworst(t) is the position of the worst rabbit in the population at time t;
α and β are two parameters that control the exploration and exploitation behavior of the algorithm.

Juvenile rabbit update equation:

X_j(t + 1) = X_j(t) + (X_a(t) − X_j(t)) × rand()(4)

where

Xj(t) is the position of the juvenile rabbit at time t;
Xa(t) is the position of the adult rabbit that the juvenile rabbit is following at time t;
rand() is a random number between 0 and 1;

The pseudo-code of this wonderful algorithm is shown below [19].

#### Pseudo-Code of Artificial Rabbits Optimization Algorithm

The pseudo-code for the artificial rabbits optimization algorithm is as follows:

Step 1: Initialize the population of rabbits (or initial solution);

Step 2: For each rabbit, calculate the fitness value by evaluating its objective function;

Step 3: Evaluate the best rabbit in the population and set it as the current best solution;

Step 4: For each rabbit in the population, delete the rabbits with the worst fitness value;

Step 5: Create new rabbits in the population by mutating the genes of the best rabbit;

Step 6: Go back to step 2 until the best solution is obtained [20].

**Figure 2:**
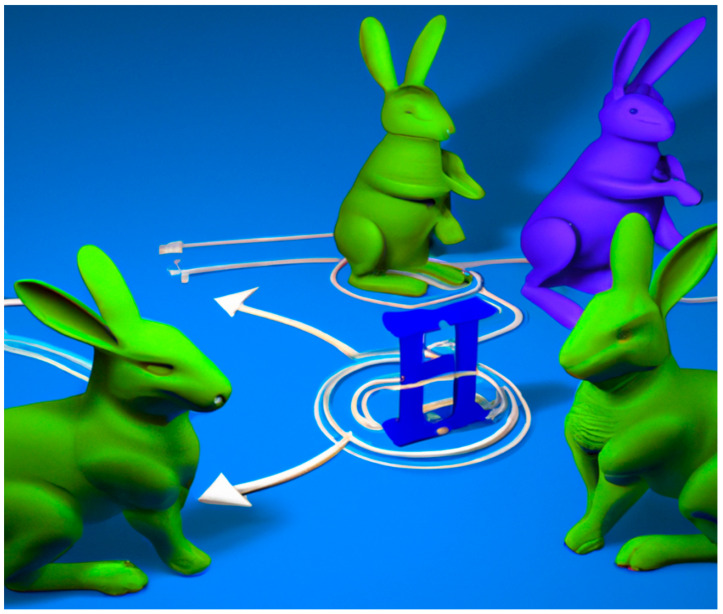
Finding best solution using artificial rabbits optimization.

Figure 2 shows finding best solution using artificial rabbits algorithm.

### 3.2. Dynamic Arithmetic Optimization Algorithm

The dynamic arithmetic optimization algorithm (DAOA) was first proposed by Professor Prabhakar Ragde and his team of researchers at the University of Waterloo in 1995 (Li et al., 2023) [21]. The algorithm is based on evolutionary optimization and has been used to solve a variety of optimization problems, ranging from scheduling and routing to portfolio optimization. The dynamic arithmetic optimization algorithm (DAOA) is a heuristic-based algorithm that uses probabilistic and stochastic search strategies to generate optimal solutions. It works by searching the solution space using a random number generator and evaluating the resulting solutions against an optimization objective. The algorithm then modifies the initial solution parameters with each iteration, gradually converging towards a local optimum. The dynamic arithmetic optimization algorithm (DAOA) has been used to solve a variety of optimization problems, such as scheduling and routing, image processing, and robotics. Additionally, it has been used for portfolio optimization and parameter optimization in machine learning and artificial intelligence models. The DAOA provides an efficient and accurate way to identify the optimal solution for a given problem.

The mathematical equations for DAOA are as follows:

Individual update equation:

X_i(t + 1) = X_i(t) + a × (X_best(t) − X_i(t)) + b × (X_i(t) − X_worst(t))(5)

where

Xi(t) is the position of the individual at time t;
Xbest(t) is the position of the best individual in the population at time t;
Xworst(t) is the position of the worst individual in the population at time t;
a and b are two parameters that control the exploration and exploitation behavior of the algorithm.

The pseudo-code of the dynamic arithmetic optimization algorithm is given below [22].

#### Pseudo-Code of Dynamic Arithmetic Optimization Algorithm

The pseudo-code for the dynamic arithmetic optimization algorithm is as follows:

Step 1: Initialize the parameters of the optimization problem;

Step 2: Generate a random initial solution;

Step 3: Evaluate the current solution;

Step 4: Iteratively modify the solution parameters;

Step 5: Check convergence criteria to decide if the algorithm has converged;

Step 6: If the algorithm has converged, stop; otherwise, go to Step 2.

**Figure 3:**
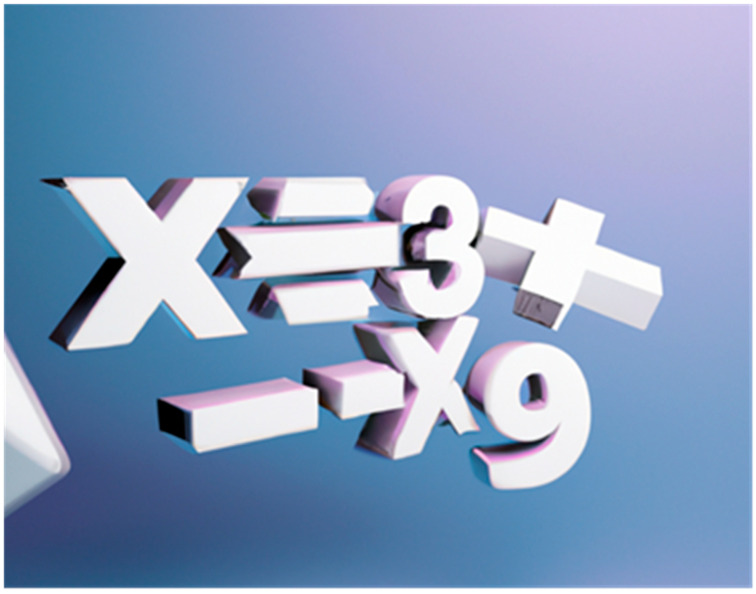
Finding best solution using dynamic arithmetic optimization algorithm.

Figure 3 shows finding best solution using dynamic arithmetic optimization algorithm.

## 4. Discussion

**Figure 4:**
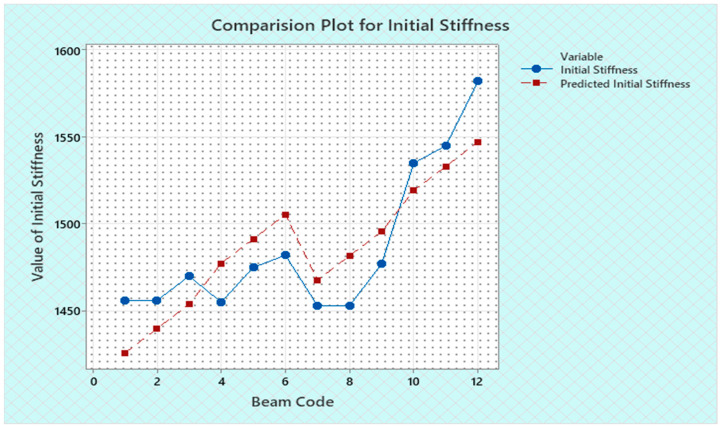
Comparison plot for initial stiffness response.

**Figure 5:**
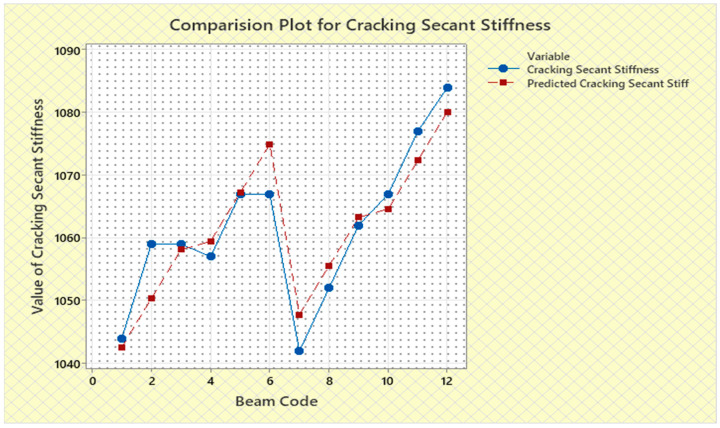
Comparison plot for cracking secant stiffness response.

**Figure 6:**
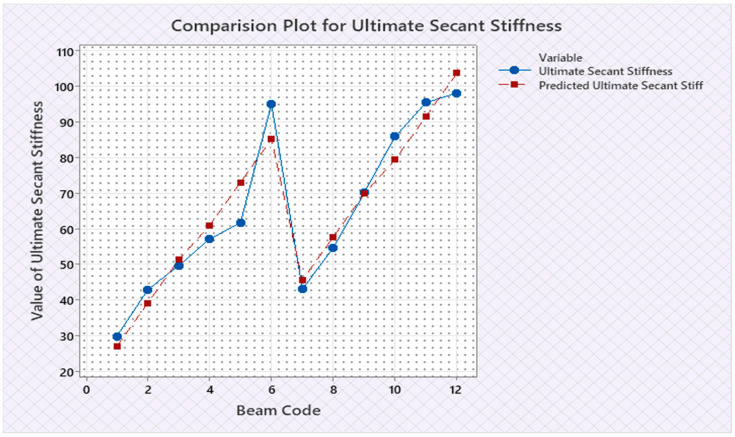
Comparison plot for ultimate secant stiffness response.

**Figure 7:**
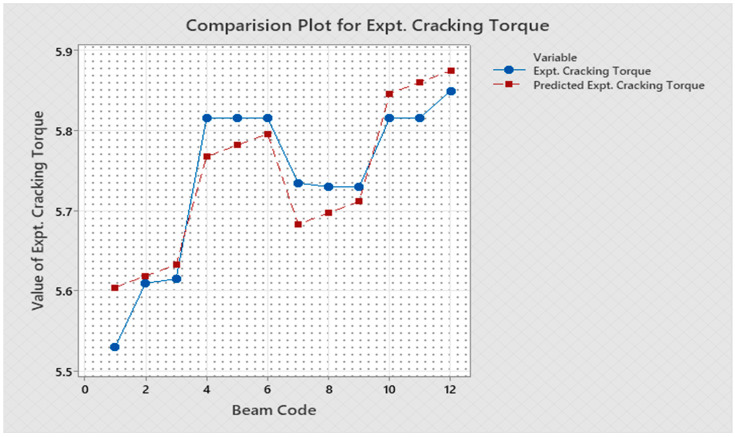
Comparison plot for experimental cracking torque response.

**Figure 8:**
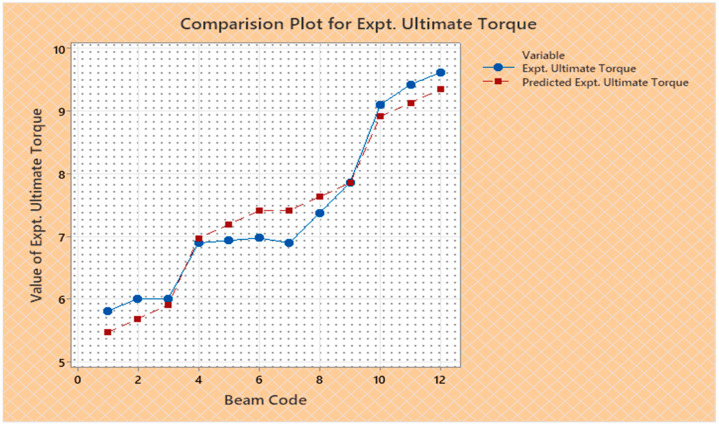
Comparison plot for experimental ultimate torque response.

**Figure 9:**
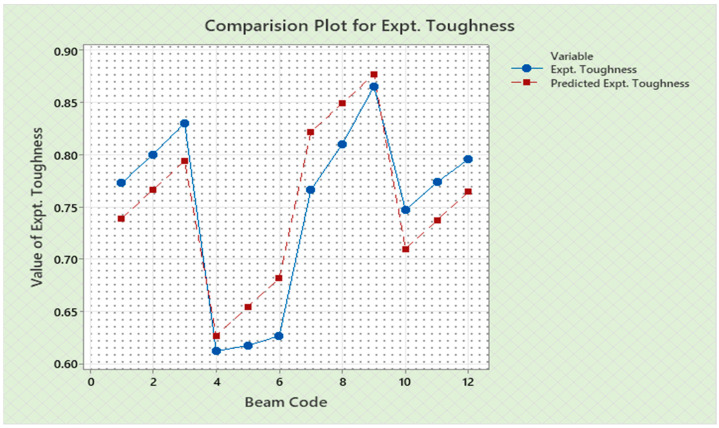
Comparison plot for experimental toughness response.

**Figure 10:**
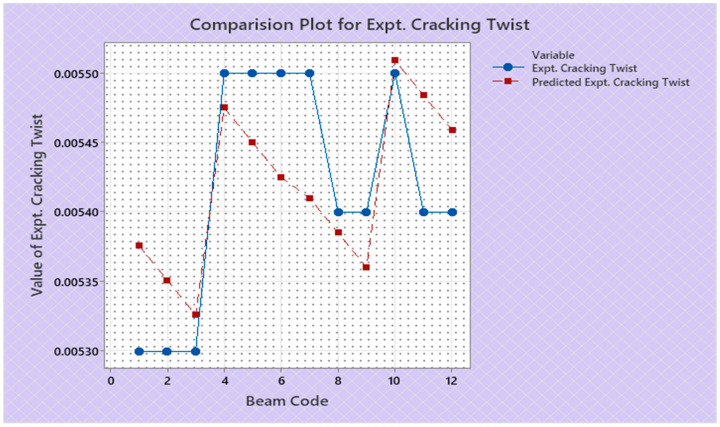
Comparison plot for experimental cracking twist response.

**Figure 11:**
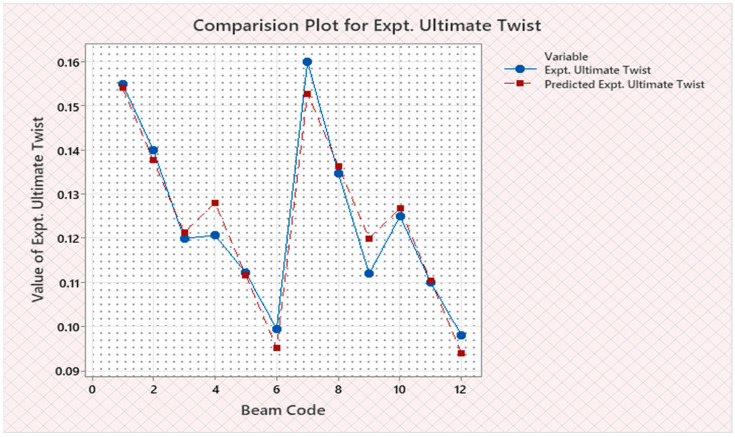
Comparison plot for experimental ultimate twist response.

**Figure 12:**
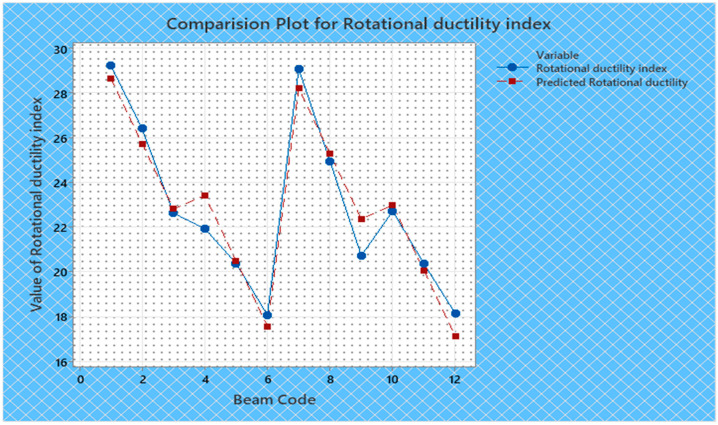
Comparison plot for rotational ductility index response.

Torque and twist obtained from experimental investigation and from predicted values at cracking and ultimate were analyzed and discussed in this section. The inputs of the torsional parameters of ferrocement “U” type wrapped beams are mentioned in Table 1. Here, four types of reinforced beams are considered: (1) U—under type, (2) L—longitudinally type, (3) T—transversely type, and (4) C—completely type. In each series of reinforced beams, there are three designations considered, such as (i) 3N, (ii) 4N, and (iii) 5N, respectively. The beam code is denoted from 1 to 12 for each reinforced beam specification. Similarly, the longitudinal steel diameter, longitudinal steel yield diameter, transverse steel diameter, transverse steel yield strength, and number of mesh layers are considered input variables and are mentioned in Table 1. After taking the values of inputs, the entire experiment is carried out, and the output responses are measured, i.e., (1) initial stiffness, (2) cracking secant stiffness, (3) ultimate secant stiffness, (4) experimental cracking torque, (5) experimental ultimate torque, (6) experimental toughness, (7) experimental cracking twist, (8) experimental ultimate twist, and (9) rotational ductility index, respectively. Following that, a linear regression analysis of each output response was performed, and it was discovered that input parameters, such as (1) beam code, (2) longitudinal steel diameter, and (3) transverse steel diameter, had a much greater effect on each output response than other input variables. The obtained regression equations are listed from Equations (6)–(14) for each output response. The regression equations are utilized to predict the nine output responses, and the obtained results are tabulated in Table 2. The comparison plot of experimental values versus predicted values of each output response is displayed in Figure 4, Figure 5, Figure 6, Figure 7, Figure 8, Figure 9, Figure 10, Figure 11 and Figure 12. From these graphs, it can be observed that the responses like ultimate secant stiffness, experimental ultimate torque, experimental ultimate twist, and rotational ductility index have a smaller margin between their experimental and predicted values.

### Torsional Behavior of Normal-Strength Beams

The torsional behavior of normal-strength beams is mainly due to their geometry and material properties. In general, the stiffness of the beam in torsion is determined by its cross-sectional shape, length, and the type of material used. Additionally, the mechanical and structural properties of the material also affect the torsional capacity of the beam. The torsional behavior of beams can be modeled using finite element analysis or experimental testing. The torque–twist responses of normal-strength concrete beams with ferrocement “U” wrap (plain beams and reinforced concrete beams) with various types were tested and analyzed [23].

**Figure 13:**
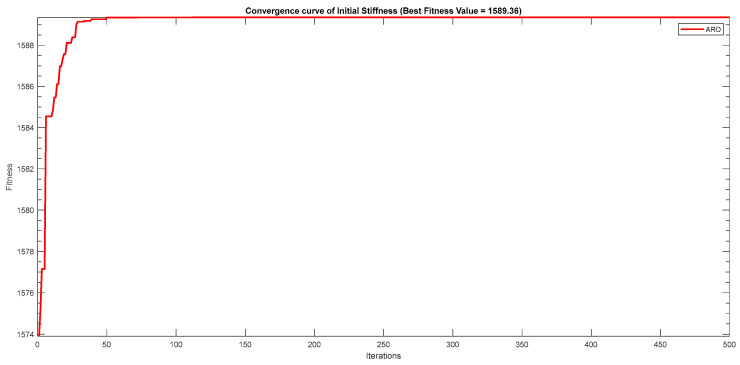
Obtained convergence plot of ARO for initial stiffness response.

**Figure 14:**
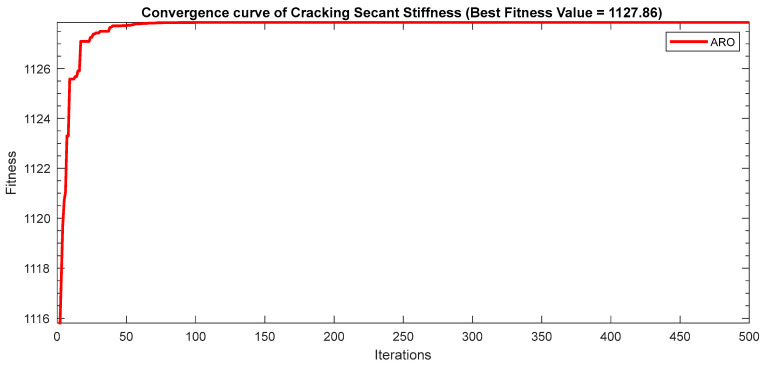
Obtained convergence plot of ARO for cracking secant stiffness response.

**Figure 15:**
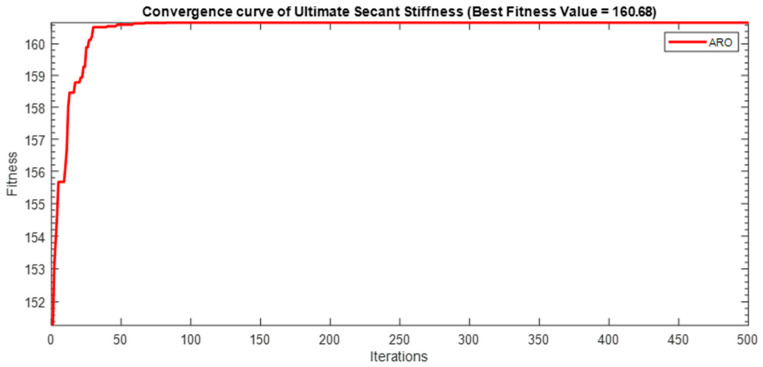
Obtained convergence Plot of ARO for ultimate secant stiffness response.

**Figure 16:**
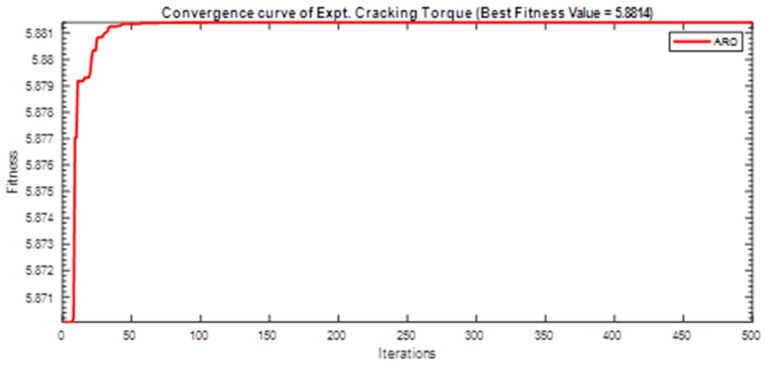
Obtained convergence plot of ARO for experimental cracking torque response.

**Figure 17:**
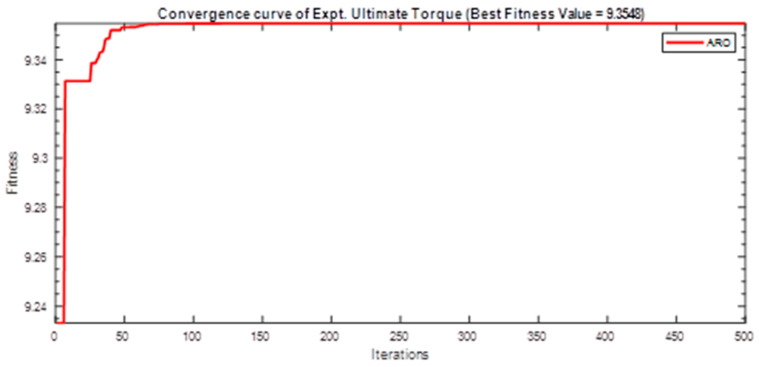
Obtained convergence plot of ARO for experimental ultimate torque response.

**Figure 18:**
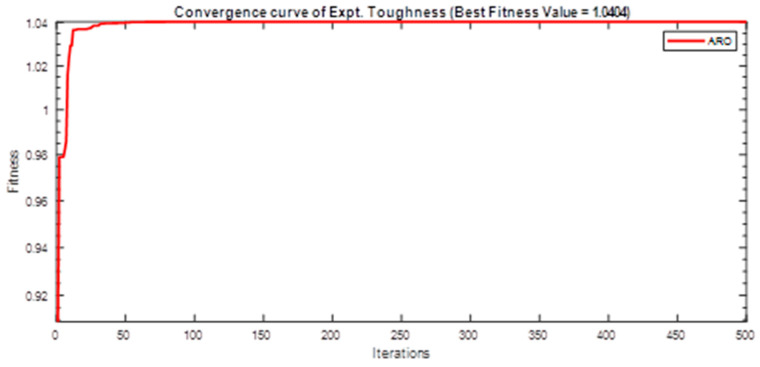
Obtained convergence plot of ARO for experimental toughness response.

**Figure 19:**
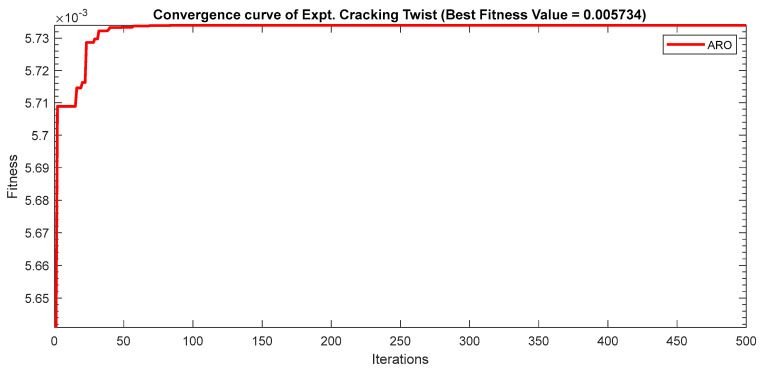
Obtained convergence plot of ARO for experimental cracking twist response.

**Figure 20:**
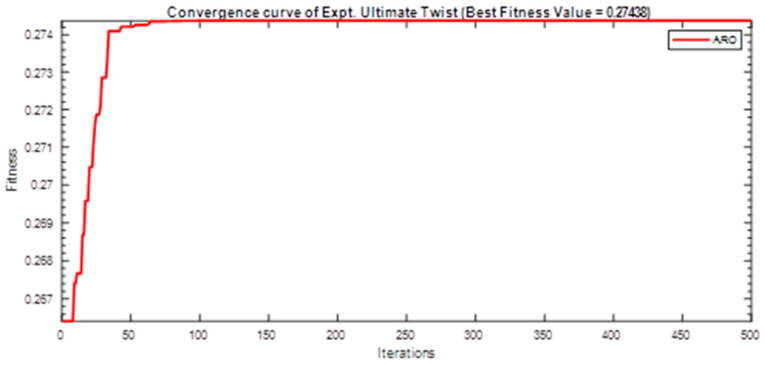
Obtained convergence plot of ARO for experimental ultimate twist response.

**Figure 21:**
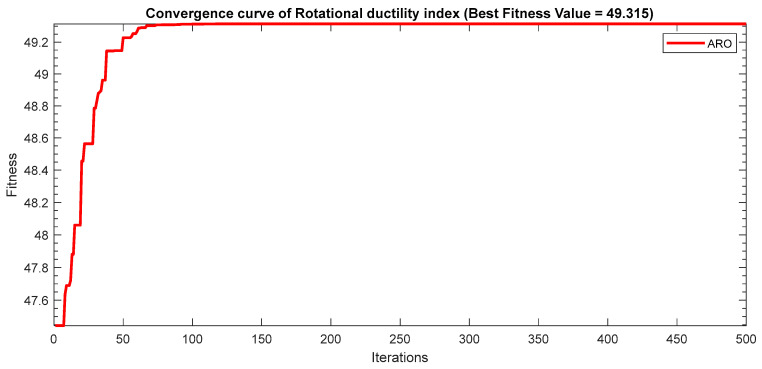
Obtained convergence plot of ARO for rotational ductility index response.

**Figure 22:**
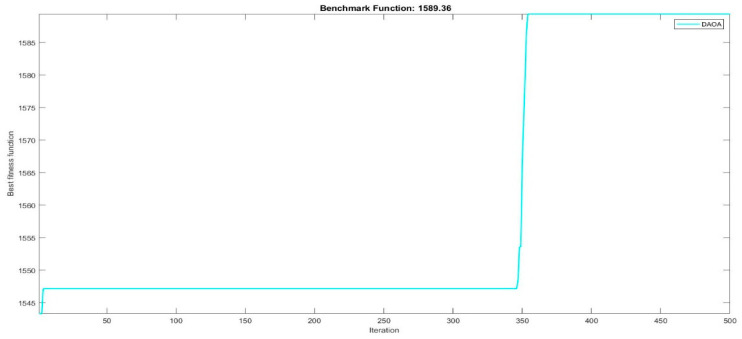
Obtained convergence plot of DAOA for initial stiffness response.

**Figure 23:**
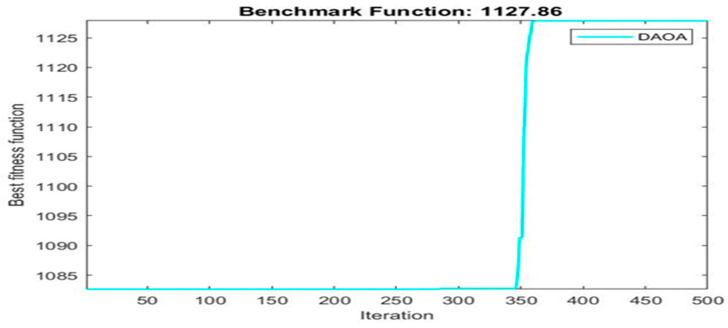
Obtained convergence plot of DAOA for cracking secant stiffness response.

**Figure 24:**
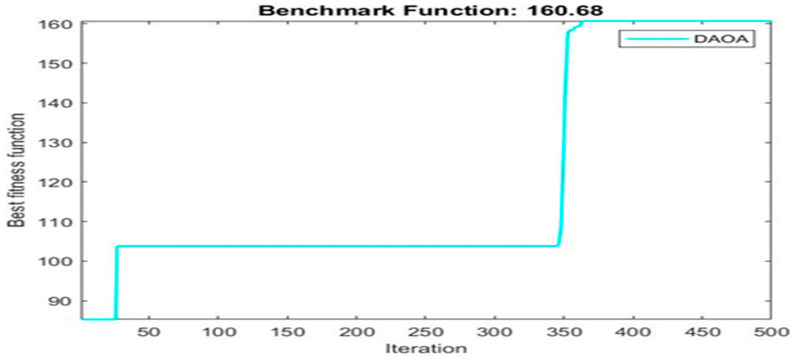
Obtained convergence plot of DAOA for ultimate secant stiffness response.

**Figure 25:**
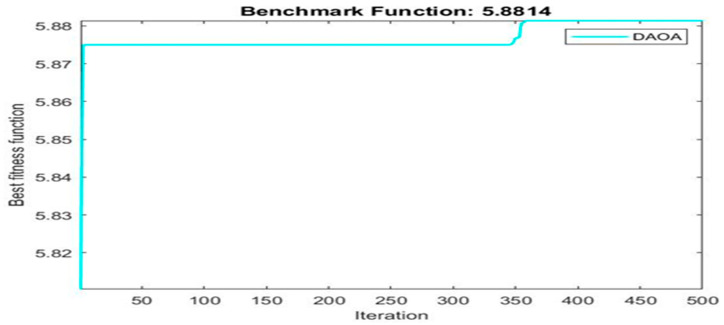
Obtained convergence plot of DAOA for experimental cracking torque response.

**Figure 26:**
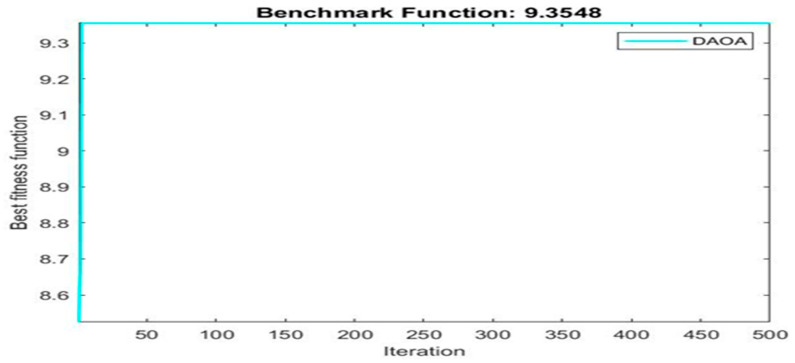
Obtained convergence plot of DAOA for experimental ultimate torque response.

**Figure 27:**
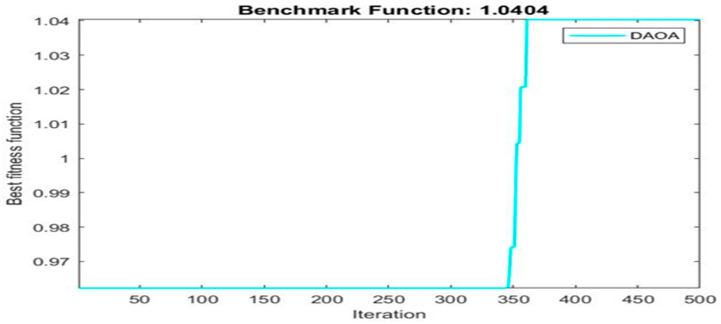
Obtained convergence plot of DAOA for experimental toughness response.

**Figure 28:**
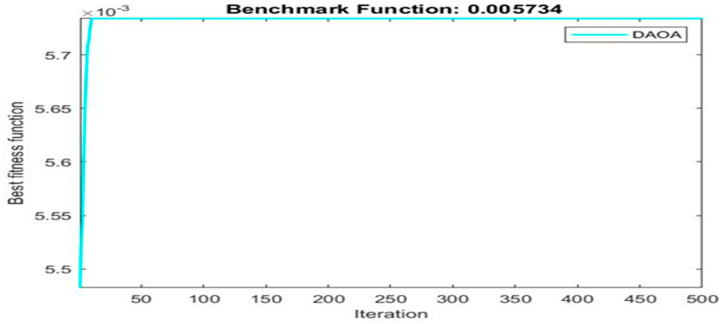
Obtained convergence plot of DAOA for experimental cracking twist response.

**Figure 29:**
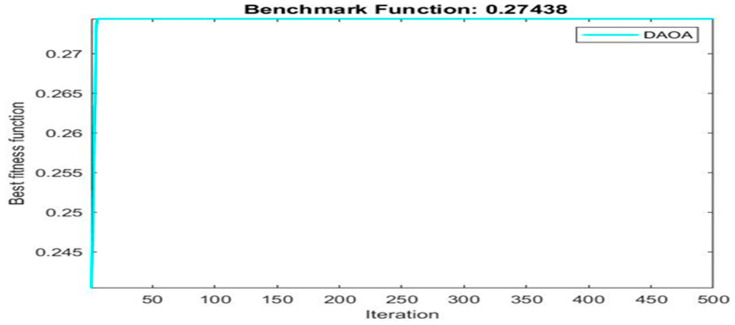
Obtained convergence plot of DAOA for experimental ultimate twist response.

**Figure 30:**
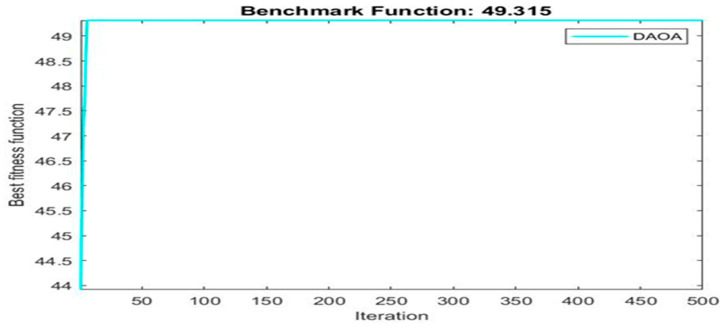
Obtained convergence plot of DAOA for rotational ductility index response.

Next, two meta-heuristic algorithms [8] are proposed to determine the global maximum point value for each response. Here, the obtained regression equations are utilized as the objective function in the optimization algorithms, and a range of significant variables, such as (1) beam code, (2) longitudinal steel diameter, and (3) transverse steel diameter, are taken as the constraints for this optimization problem. The entire simulation of two optimization algorithms was carried out in Matlab 2018 on a computer with 4 GB of RAM and an Intel Core i5 processor running Microsoft Windows. The convergence plots of each response in the above-mentioned two algorithms are displayed in Figure 13, Figure 14, Figure 15, Figure 16, Figure 17, Figure 18, Figure 19, Figure 20, Figure 21, Figure 22, Figure 23, Figure 24, Figure 25, Figure 26, Figure 27, Figure 28, Figure 29 and Figure 30 with the best fitness value. In this analysis, the number of iterations for each simulation is kept constant, and the corresponding value is 500. Furthermore, the proposed optimization approach’s criteria consider the maximization problem type for each output response with the single-objective function-type optimization problem. From all the plots of convergence, it can be observed that the curve of fitness function is converging in an upward direction after 100 iterations in the case of the ARO-type algorithm, whereas the convergence of fitness values occurs after 400 iterations in the case of the DAOA-type algorithm for all nine output responses. From this optimization analysis, it can be observed that a minimum number of iterations is required to find the global optimum in the case of an ARO-type algorithm. Similarly, the time of execution of the ARO-type algorithm was found to be minimal as compared to the DAOA-type algorithm. Finally, the obtained results of the global optimum value, along with their corresponding optimal settings of input variables from two proposed nature-based optimization algorithms, are tabulated in Table 3. Here, it can be observed that all the results obtained for nine outputs are the same in both algorithms.

**Objective Functions**

Initial stiffness = 1529 + 14 × (A) + 1.58 × (B) − 21.1 × (C)(6)

Cracking secant stiffness = 1165.3 + 7.75 × (A) − 1.07 × (B) − 20.67 × (C)(7)

Ultimate secant stiffness = 180.6 + 12.14 × (A) − 0.43 × (B) − 27.17 × (C)(8)

Expt. cracking torque = 5.489 + 0.0142 × (A) + 0.0201 × (B) − 0.0032 × (C)(9)

Expt. ultimate torque = 2.53 + 0.219 × (A) + 0.1404 × (B) + 0.314 × (C)(10)

Expt. toughness = 1.151 + 0.0274 × (A) − 0.03234 × (B) − 0.0409 × (C)(11)

Expt. cracking twist = 0.004675 − 0.000025 × (A) + 0.000029 × (B) + 0.000092 × (C)(12)

Expt. ultimate twist = −0.1441 − 0.0164 × (A) + 0.00386 × (B) + 0.04857 × (C)(13)

Rotational ductility index = −23.24 − 2.925 × (A) + 0.59 × (B) + 8.55 × (C)(14)

A, B, and C represent beam code, long. steel diameter, and trans. steel diameter, respectively.

The constraint values of beam code, long. steel diameter, and trans. steel diameter:

lower Limits = [1 6 6] and upper limits = [12 12 8].

To obtain the torsional strength of ferrocement “U” wrapped beams effectively, the following steps can be followed:

- Design of the beam: the beam should be designed to ensure that it has the desired geometry and reinforcement pattern, which is critical for torsional strength.
- Material properties: the properties of the materials used in the beam, such as the type and quality of cement, reinforcement steel, and mortar, should be known and considered.
- Load testing: The beam should be subjected to a series of load tests to determine its torsional strength. This can be carried out using a universal testing machine, where the beam is subjected to a torsional load until failure.
- Data collection and analysis: the load–displacement data from the load tests should be collected and analyzed to determine the torsional strength of the beam.
- Finite element analysis: Finite element analysis (FEA) can be used to model the behavior of the beam under torsional loading, providing an accurate estimate of its torsional strength. This can be compared to the results obtained from load testing to validate the FEA results.
- Comparison with theoretical models: the torsional strength of the beam can be compared to theoretical models, such as Timoshenko’s theory, to determine its accuracy.

Lastly, to effectively determine the torsional strength of ferrocement “U” wrapped beams, a combination of experimental testing and numerical simulations should be used.

According to the findings of this research work, reinforced concrete beams’ torsional strength is greatly increased by ferrocement “U” wrapping. This result is in line with those of other research studies like Behera (2018) [23] and Obaidat (2020) [8]. In each of these investigations, it was discovered that ferrocement “U” wrapping significantly enhanced the ultimate torque and breaking of beams by up to 98%. The grade of core concrete, the number of mesh layers, and the reinforcing ratio all had an impact on the torsional strength of ferrocement “U” wrapped beams, according to the present research. This conclusion is in line with those of earlier research as well. For instance, Behera (2018) [23] discovered that when the grade of core concrete and the reinforcing ratio enhanced, the torsional strength of ferrocement “U” wrapped beams also increased [23].

The torsional strength of ferrocement “U” wrapped beams, however, was not observed to be influenced by the number of mesh layers, according to the present research. In contrast, Obaidat (2020) [8] discovered that the number of mesh layers enhanced the torsional strength of ferrocement “U” wrapped beams. The differing experimental techniques utilized in the two investigations may be the cause of this discrepancy in results [8].

Finally, the present study’s findings and those of earlier research on the torsional strength of ferrocement “U” wrapped beams are in excellent accord. The present study’s findings add to the body of data supporting the notion that ferrocement “U” wrapping is a method with promise for boosting the torsional strength of reinforced concrete beams.

Ćirović (2014) [24] focuses on the optimization of ultra-high performance fiber reinforced concrete (UHPFRC) beams using genetic algorithms. Li (2021) [25] presents a structural optimization study of the main beam of an ultra-low clearance hoisting system using an improved particle swarm algorithm. Silva (2017) [26] and Silva (2018) [27] both discuss the structural optimization of internally reinforced beams using a finite element updating code built in MATLAB.

## 5. Novel Contributions of the Study

This research introduces several novel contributions to the field of reinforced concrete beams with ferrocement “U” wraps. Firstly, our study offers a comprehensive investigation into the torsional behavior of beams subjected to various states of torsion. This includes an in-depth analysis of factors such as the grade of core concrete, number of mesh layers, and reinforcing ratio, shedding light on their influence on torsional strength. Additionally, our experimentation reveals a notable increase in cracking and ultimate torque in beams with ferrocement wraps compared to unwrapped counterparts. Notably, we observed a remarkable 98% enhancement in maximum ultimate torque for over-reinforced beams. Furthermore, our study provides valuable insights into the impact of different reinforcement configurations within the core concrete on torsional behavior. Through a combination of experimental testing and advanced numerical simulations, we establish a reliable method for determining the torsional strength of ferrocement “U” wrapped beams. These findings collectively contribute to the growing body of knowledge supporting the efficacy of ferrocement wrapping in bolstering the torsional strength of reinforced concrete beams.

## 6. Conclusions

The authors conducted an experiment to determine the best way to measure how strong a beam made of ferrocement is when it is twisted. They used different designs and materials to make the beam and tested it after 28 days. They found that the iron made the beam stronger when it was twisted. In conclusion, the best way to determine the torsional strength of ferrocement “U” wrapped beams is through the casting of prototype beams and testing them under torsional loading. Variations in the design, such as the number of mesh layers, reinforcement in the core concrete, and the grade of the concrete and mortar used, were taken into consideration during the experiment. The beams were cast with different states of torsion and tested after 28 days of curing. The results were obtained through experimental investigation as well as through analytical and soft computing methods. The following conclusions were made considering the findings:

- Experimental testing is the most reliable method for validating the results of numerical simulations.
- The ferrocement wrapping increases the cracking and ultimate torque of beams with respect to unwrapped beams of the same dimension.
- Regardless of the number of mesh layers, the cracking torque for a certain grade of core concrete and ferrocement “U” wrap is almost the same for all states of torsion.
- Due to an increase in the shear strength of a higher grade of material in the peripheral, cracking torque rises as the grade of core concrete and mortar in the ferrocement wrap increases.
- The breaking torque of the ferrocement “U” wrapped beams was comparable to that of the unwrapped control beams.
- Maximum ultimate torque was noticed for completely over-reinforced beams and found to be 98% greater than that of the control specimen.
- The quality of the core concrete and mortar in the ferrocement wrap rises, increasing ultimate torque.
- The cracking and ultimate torque of ferrocement “U” wrapped beams with single-type reinforcement in the core concrete were similar to those of plain beams with “U” wrap.
- Regardless of torsion states, M35 grade concrete “U” wrap beams have a twist at cracking torque that is almost the same.
- Maximum twist was found under reinforced beams of M35 grade concrete, as stiffness at ultimate torque is low.
- The only kind of beam that has experienced higher twists than transversely reinforced beams was under-reinforced beams.

## Figures and Tables

**Table 1:** Inputs of torsional parameters of ferrocement “U” wrapped beams.

Beam Code	Series	Designation	Beam Code	Long. Steel Diameter	Long. Steel Yield Strength	Trans. Steel Diameter	Trans. Steel Yield Strength	No. of Mesh Layers
1	U—Under-reinforced beams	U3N	1	6	350	6	350	3
2	U—Under-reinforced beams	U4N	2	6	350	6	350	4
3	U—Under-reinforced beams	U5N	3	6	350	6	350	5
4	L—Longitudinally over-reinforced beams	Lo3N	4	12	440	6	350	3
5	L—Longitudinally over-reinforced beams	Lo4N	5	12	440	6	350	4
6	L—Longitudinally over-reinforced beams	Lo5N	6	12	440	6	350	5
7	T—Transversely over-reinforced beams	To3N	7	6	350	8	465	3
8	T—Transversely over-reinforced beams	To4N	8	6	350	8	465	4
9	T—Transversely over-reinforced beams	To5N	9	6	350	8	465	5
10	C—Completely over-reinforced beams	Co3N	10	12	440	8	465	3
11	C—Completely over-reinforced beams	Co4N	11	12	440	8	465	4
12	C—Completely over-reinforced beams	Co5N	12	12	440	8	465	5

**Table 2:** Experimental and predicted outputs of torsional parameters of ferrocement “U” wrapped beams.

Sl. No.	Beam Code	Initial Stiffness	Predicted Initial Stiffness	Cracking Secant Stiffness	Predicted Cracking Secant Stiffness	Ultimate Secant Stiffness	Predicted Ultimate Secant Stiffness	Expt. Cracking Torque	Predicted Expt. Cracking Torque	Expt. Ultimate Torque	Predicted Expt. Ultimate Torque	Expt. Toughness	Predicted Expt. Toughness	Expt. Cracking Twist	Predicted Expt. Cracking Twist	Expt. Ultimate Twist	Predicted Expt. Ultimate Twist	Rotational Ductility Index	Predicted Rotational Ductility Index
U3N	1	1456	1425.88	1044	1042.61	30	27.14	5.53	5.6046	5.816	5.4754	0.7726	0.73896	0.0053	0.005376	0.155	0.15408	29.25	28.675
U4N	2	1456	1439.88	1059	1050.36	42.97	39.28	5.61	5.6188	6.01	5.6944	0.8	0.76636	0.0053	0.005351	0.14	0.13768	26.42	25.75
U5N	3	1470	1453.88	1059	1058.11	49.8	51.42	5.615	5.633	6.01	5.9134	0.83	0.79376	0.0053	0.005326	0.12	0.12128	22.64	22.825
Lo3N	4	1455	1477.36	1057	1059.44	57.15	60.98	5.816	5.7678	6.899	6.9748	0.6126	0.62712	0.0055	0.005475	0.1207	0.12804	21.95	23.44
Lo4N	5	1475	1491.36	1067	1067.19	61.84	73.12	5.816	5.782	6.939	7.1938	0.6177	0.65452	0.0055	0.00545	0.1122	0.11164	20.4	20.515
Lo5N	6	1482	1505.36	1067	1074.94	95.09	85.26	5.816	5.7962	6.979	7.4128	0.627	0.68192	0.0055	0.005425	0.0995	0.09524	18.09	17.59
To3N	7	1453	1467.68	1042	1047.77	43.11	45.64	5.735	5.6834	6.899	7.4174	0.766	0.82156	0.0055	0.00541	0.16	0.15282	29.09	28.225
To4N	8	1453	1481.68	1052	1055.52	54.75	57.78	5.73	5.6976	7.38	7.6364	0.81	0.84896	0.0054	0.005385	0.1348	0.13642	24.96	25.3
To5N	9	1477	1495.68	1062	1063.27	70.19	69.92	5.73	5.7118	7.86	7.8554	0.865	0.87636	0.0054	0.00536	0.112	0.12002	20.74	22.375
Co3N	10	1535	1519.16	1067	1064.6	85.95	79.48	5.816	5.8466	9.105	8.9168	0.7469	0.70972	0.0055	0.005509	0.125	0.12678	22.73	22.99
Co4N	11	1545	1533.16	1077	1072.35	95.5	91.62	5.816	5.8608	9.426	9.1358	0.7734	0.73712	0.0054	0.005484	0.11	0.11038	20.37	20.065
Co5N	12	1582	1547.16	1084	1080.1	98.23	103.76	5.85	5.875	9.62	9.3548	0.7957	0.76452	0.0054	0.005459	0.098	0.09398	18.15	17.14

**Table 3:** Obtained results of two meta-heuristic algorithms.

Optimization Technique	Global Optimum Value	Optimum Value of Beam Code	Optimum Value of Long. Steel Diameter	Optimum Value of Trans. Steel Diameter
ARO	1589.36	12	12	6
	1127.86	12	6	6
	160.68	12	6	6
	5.8814	12	12	6
	9.3548	12	12	8
	1.0404	12	6	6
	0.005734	1	12	8
	0.27438	1	12	8
	49.315	1	12	8
DAOA	1589.36	12	12	6
	1127.86	12	6	6
	160.68	12	6	6
	5.8814	12	12	6
	9.3548	12	12	8
	1.0404	12	6	6
	0.005734	1	12	8
	0.27438	1	12	8
	49.315	1	12	8

## Data Availability

Not applicable.

## Nomenclature

e	effective parameter involving beam code, long. steel diameter, and trans. steel diameter
A	beam code
B	longitudinal steel diameter
C	transverse steel diameter
Expt.	experimental
ARO	artificial rabbits optimization
DAOA	differential ant colony optimization algorithm
FEA	finite element analysis
M20	grade of concrete with 20 MPa compressive strength
M25	grade of concrete with 25 MPa compressive strength
M30	grade of concrete with 30 MPa compressive strength
Long. steel diameter	diameter of longitudinal reinforcement
Trans. steel diameter	diameter of transverse reinforcement
Beam code	type of beam (e.g., rectangular, I-beam, etc.)
Cracking torque	torque at which the first crack appears
Ultimate torque	maximum torque that the beam can withstand before failure
Cracking twist	angle of twist at cracking torque
Ultimate twist	angle of twist at ultimate torque
Rotational ductility index	measure of the beam’s ability to deform plastically before failure

## References

[1] Deifalla A., Awad A., Elgarhy M. (2013). Effectiveness of externally bonded CFRP strips for strengthening flanged beams under torsion: An experimental study. J. Eng. Struct..

[2] Dong Z.Q., Wu G., Xu B., Wang X., Taerwe L. (2018). Bond performance of alkaline solution pre-exposed FRP bars with concrete. Mag. Concr. Res..

[3] Tahir M., Wang Z.U., Ali K.M. (2019). Axial compressive behavior of square concrete columns confined with CFRP strip ties using wet lay-up technique. Constr. Build. Mater..

[4] Karayannis C.G., Chalioris C.E., Sirkelis G.M. (2008). Local retrofit of exterior RC beam-column joints using thin RC jackets—An experimental study. Earthq. Eng. Struct. Dyn..

[5] Isleem H.F., Wang D., Wang Z. (2018). Modeling the axial compressive stress-strain behavior of CFRP-confined rectangular RC columns under monotonic and cyclic loading. Compos. Struct..

[6] Pancharam S., Belarbi A. Torsional behavior of reinforced concrete beams strengthened with FRP Composites. Proceedings of the First FIB Congress.

[7] Behera G.C. (2018). A model to predict the torsional stiffness of ‘U-wrapped’ reinforced concrete beams. Proc. Inst. Civ. Eng. Struct. Build..

[8] Yaseen T.O., Ashteyat A.M., Obaidat A.T. (2020). Performance of RC beam strengthened with NSM-CFRP strip under pure torsion: Experimental and numerical study. Int. J. Civ. Eng..

[9] Chalioris C.E. (2008). Torsional strengthening of rectangular and flanged beams using carbon fiber-reinforced-polymers—Experimental study. Constr. Build. Mater..

[10] Li B., Lam E.S.S., Wu B., Wang Y.Y. (2013). Experimental investigation on reinforced concrete interior beam-column joints rehabilitated by ferrocement jackets. Eng. Struct..

[11] ACI Committee (1979). Ferrocement-Materials and Applications.

[12] (1997). ACI-549 Committee Report R-97. State of Art Report on Ferrocement.

[13] Shannag M.J., Mourad S.M. (2012). Flowable high strength cementitious matrices for ferrocement applications. Constr. Build. Mater..

[14] Chalioris C.E., Thermou G.E., Pantazopoulou S.J. (2014). Behaviour of rehabilitated rc beams with self-compacting concrete jacketing—Analytical model and test results. Constr. Build. Mater..

[15] Behera G.C., Rao T.D.G., Rao C.B.K. (2014). Study of Post-Cracking Torsional Behaviour of High-Strength Reinforced Concrete Beams with a Ferrocement Wrap. Slovak J. Civ. Eng..

[16] Zhang W., Zhang R., Wu C., Goh A.T.C., Lacasse S., Liu Z., Liu H. (2020). State-of-the-art review of soft computing applications in underground excavations. Geosci. Front..

[17] Ajay S.S., Masuku M.B. (2013). Applications of Modeling and Statistical Regression Techniques in Research. Res. J. Math. Stat. Sci..

[18] Wang L., Cao Q., Zhang Z., Mirjalili S., Zhao W. (2020). Artificial Rabbits Optimization: A new bio-inspired meta-heuristic algorithm for solving engineering optimization problems. Eng. Appl. Artif. Intell..

[19] Maher M., Abdel A.S.H., Ibrahim A.M., El-Shahat A. (2023). Novel Mathematical Design of Triple-Tuned Filters for Harmonics Distortion Mitigation. Energies.

[20] Mori H., Tani H. (2002). Two-staged tabu search for determining optimal allocation of D-FACTS in radial distribution systems with distributed generation. IEEE/PES Transm. Distrib. Conf. Exhib..

[21] Li X.D., Wang J.S., Hao W.K., Song H.M., Zhao X.R. (2023). Lorentz chaotic trigonometric function pedigree based arithmetic optimization algorithm. J. Intell. Fuzzy Syst..

[22] Khodadadi N., Snasel V., Mirjalili S. (2022). Dynamic arithmetic optimization algorithm for truss optimization under natural frequency constraints. IEEE Access.

[23] Behera G.C., Dhal M. (2018). Torsional behaviour of normal strength RCC beams with ferrocement “U” wraps. Facta Univ. Ser. Archit. Civ. Eng..

[24] Ćirović G., Radonjanin V., Trivunić M., Nikolić D. (2014). Optimization of UHPFRC beams subjected to bending using genetic algorithms. J. Civ. Eng. Manag..

[25] Li Z., Gao Y. Structural Optimization of Main Beam of Ultra-Low Clearance Hoisting System for EHV Cables. Proceedings of the 10th International Conference on Informatics, Environment, Energy and Applications.

[26] Silva H.M., Meireles J.F. (2017). Structural Optimization of Internally Reinforced Beams Subjected to Uncoupled and Coupled Bending and Torsion Loadings for Industrial Applications. Mech. Mech. Eng..

[27] Silva H.M., de Meireles J.F.B. (2018). Design Optimisation of Internally Reinforced Beams Subjected to Bending Loading. Advanced Engineering Forum.

